# Deciphering the Relationship Between Free and Vesicular Hemoglobin in Stored Red Blood Cell Units

**DOI:** 10.3389/fphys.2022.840995

**Published:** 2022-02-08

**Authors:** Vassilis L. Tzounakas, Alkmini T. Anastasiadi, Marilena E. Lekka, Effie G. Papageorgiou, Konstantinos Stamoulis, Issidora S. Papassideri, Anastasios G. Kriebardis, Marianna H. Antonelou

**Affiliations:** ^1^Section of Cell Biology and Biophysics, Department of Biology, School of Science, National and Kapodistrian University of Athens (NKUA), Athens, Greece; ^2^Laboratory of Biochemistry, Department of Chemistry, University of Ioannina, Ioannina, Greece; ^3^Laboratory of Reliability and Quality Control in Laboratory Hematology (HemQcR), Department of Biomedical Sciences, School of Health and Welfare Sciences, University of West Attica (UniWA), Egaleo, Greece; ^4^Hellenic National Blood Transfusion Centre, Athens, Greece

**Keywords:** hemolysis, red blood cells, storage lesion, extracellular vesicles, size distribution

## Abstract

Red blood cells (RBCs) release hemoglobin (Hb)-containing extracellular vesicles (EVs) throughout their lifespan in the circulation, and especially during senescence, by spleen-facilitated vesiculation of their membrane. During *ex vivo* aging under blood bank conditions, the RBCs lose Hb, both in soluble form and inside EVs that accumulate as a part of storage lesion in the supernatant of the unit. Spontaneous hemolysis and vesiculation are increasingly promoted by the storage duration, but little is known about any physiological linkage between them. In the present study, we measured the levels of total extracellular and EV-enclosed Hb (EV-Hb) in units of whole blood (*n* = 36) or packed RBCs stored in either CPDA-1 (*n* = 99) or in CPD-SAGM additive solution (*n* = 46), in early, middle, and late storage. The spectrophotometry data were subjected to statistical analysis to detect possible correlation(s) between storage hemolysis and EV-Hb, as well as the threshold (if any) that determines the area of this dynamic association. It seems that the percentage of EV-Hb is negatively associated with hemolysis levels from middle storage onward by showing low to moderate correlation profiles in all strategies under investigation. Moreover, 0.17% storage hemolysis was determined as the potential cut-off, above which this inverse correlation is evident in non-leukoreduced CPDA units. Notably, RBC units with hemolysis levels > 0.17% are characterized by higher percentage of nanovesicles (<100 nm) over typical microvesicles (100–400 nm) compared with the lower hemolysis counterparts. Our results suggest an ordered loss of Hb during RBC accelerated aging that might fuel targeted research to elucidate its mechanistic basis.

## Introduction

Extracellular vesicles (EVs) are membrane-limited nanoparticles that are produced by almost every cell under homeostatic or pathological conditions. Their cargo and surface molecules depend on the type and physiological state of the parental cell. EVs contribute to significant biological processes in the erythroid lineage, including reticulocyte maturation and red blood cell (RBC) senescence. Mature RBCs have a lifespan of 120 days, during which, and due to the finite nature of their proteome, they accumulate oxidized molecules, lose enzymatic activities and present membrane/cytoskeletal defects (Ciana et al., 2017). In order to remove damaged and potentially toxic molecules, RBCs sacrifice part of their membrane to load them inside vesicles (Antonelou and Seghatchian, 2016). Since hemoglobin (Hb) is the main cytosolic protein of RBCs, it is one of the most prevalent molecules found in RBC-derived EVs (Thangaraju et al., 2020) and it has been suggested that defects in Hb might be one of the primary triggers for vesiculation (Leal et al., 2018). This is supported by the elimination of hemichromes or the redox active ferryl Hb (Welbourn et al., 2017) via microvesicles in the case of thalassemic patients (Ferru et al., 2014), or oxidatively challenged RBCs (Sudnitsyna et al., 2020), respectively.

Several RBC aging phenotypes are evident under storage. Generation of EVs is an important homeostasis mechanism which helps RBCs dispose oxidized or toxic molecules that accumulate over storage time. While extremely beneficial and “life-saving” at first, excessive shedding of membrane parts inevitably leads to less deformable (McVey et al., 2020) and morphologically altered RBCs that are more prone to lysis (Safeukui et al., 2012). Several pieces of evidence support the hypothesis that deregulation of metabolism, both energy and redox, in stored RBCs, is associated with the production of EVs during the early period of storage, while the disruption of the membrane and cytoskeleton could take the blame for the vesiculation later on (Bosman et al., 2008; Freitas Leal et al., 2020; Tzounakas et al., 2021c).

The release of free Hb and Hb-containing EVs are major aspects of storage lesion (Kim-Shapiro et al., 2011). Hemolysis and vesiculation are both affected by the storage strategy followed, as well as the storage duration (Wannez et al., 2019; Noulsri et al., 2021) and moreover, these two phenomena seem to be inter-correlated in both stored (Almizraq et al., 2018), and diseased RBCs (Olatunya et al., 2019). Nonetheless, apart from a study showing that at least during the first 21 days of storage most of the extracellular hemoglobin is encapsulated in EVs (Greenwalt et al., 1991), to our knowledge the specific spatiotemporal modal of Hb loss (soluble or vesicle encapsulated) during storage has not been studied. Thus, the aim of the present study was (a) to decipher if there is an association between the storage hemolysis levels and the percentage of Hb located inside vesicles under different storage strategies and durations, as well as (b) to find the threshold (if any) that determines the area of this dynamic association.

## Materials and Methods

### Blood Processing

We performed a correlation analysis between storage hemolysis and the percentage of vesicle-enclosed Hb in stored RBCs. For this purpose, blood samples from 181 eligible donors (with Hb > 13.5 g/dL for men or >12.5 g/dL for women) were analyzed during cold storage under the following conditions: whole blood units stored in citrate-phosphate-dextrose-adenine (CPDA-1) (*n* = 36), non-leukodepleted RBC concentrates in CPDA-1 (*n* = 99), and leukoreduced RBC concentrates in citrate-phosphate-dextrose/saline-adenine-glucose-mannitol (CPD/SAGM) preservative/additive solution (*n* = 46). The hematological data and storage-lesion profiles of the majority of those blood and RBC units have been previously reported (Tzounakas et al., 2016, 2017, 2021a,^2022^). The study has been submitted and approved by the Research Bioethics and BioSecure Committee of the Department of Biology, NKUA. Investigations were carried out in accordance with the principles of the Declaration of Helsinki.

### Hemolysis Measurements

In-bag hemolysis was calculated by measuring the levels of free Hb in the supernatant of the blood units using Harboe’s method. Briefly, each sample was centrifuged at 1,000 × *g* for 10 mins and the collected supernatant was processed again under the same conditions. The supernatant was then diluted in distilled water and incubated at room temperature for 30 mins, before measuring the absorbance at 380, 415, and 450 nm. Allen’s correction was used to calculate the concentration of released Hb, as follows: Hb(mg/dL) = (167.2 × A415 − 83.6 × A380 − 83.6 × A450) × 1/1,000 × Dilution factor × 100. Finally, the hemolysis percentage was calculated using the formula: %hemolysis = [*supernatantHb*(mg/dL)×(100−%*Hct*)]/*totalHb*(mg/dL) where Hct stands for hematocrit.

To assess the percentage of Hb released through vesicles an additional step was performed in the abovementioned protocol. An aliquot of the supernatant was centrifuged at 30,000 × *g* for 10 mins (4°C), the pellet was disposed and the levels of free Hb of the supernatant were measured following the same procedure. The percentage of vesicle-enclosed Hb was calculated by the following formula: EV-Hb(%) = [(supernatantHb(mg/dL)−*supernatantHbaftercentrifugation*(mg/dL))/*supernatantHb*(mg/dL)]×100.

### Extracellular Vesicles Evaluation by Dynamic Light Scattering

For selected samples (*n* = 10) an aliquot of the supernatant was filtered through sterile 0.8-mm pore size syringe-driven nitrocellulose filter units (Millipore, Carrigtwohill, County Cork, Ireland), before ultracentrifugation at 30,000 × *g* for 1 h at 4°C. These conditions are commonly used to isolate microvesicles of 100–800 nm, so the range of EVs studied in this research (100–450 nm) is included. Smaller EVs falling in the size range of “exosomes,” namely of 60–100 nm, may also co-precipitate (Kriebardis et al., 2008). For EV sizing, a high performance two angle particle and molecular size analyzer (Zetasizer Nano ZS, Malvern, Malvern, United Kingdom) was used, as previously reported (Tzounakas et al., 2018). The instrument operated at a wavelength of 633 nm and was equipped with a helium-neon laser at the standard angle of 173° and a glass cuvette with square aperture. The vesicle solutions were prepared in a dilute concentration approximately equal to 0.1% (wt/wt).

### Statistical Analysis

Correlations between storage hemolysis and vesicular Hb were evaluated by the Pearson’s test following testing of the variables for normal distribution and the presence of outliers (Shapiro–Wilk, Kolmogorov–Smirnov tests and detrended normal Q–Q plots). Pearson’s test is highly sensitive to outliers, thus such values were excluded, and the analysis was performed again, to minimize the false discovery rate associated with the small size of our subgroups. If the outcome was not modified, the outlier was included back to the subgroup. Between groups differences were analyzed by independent t-test. All statistical analyses were performed using the statistical package SPSS Version 22.0 (IBM Hellas, Athens, Greece, administered by NKUA). Significance was accepted at *p* < 0.05.

## Results

**FIGURE 1:**
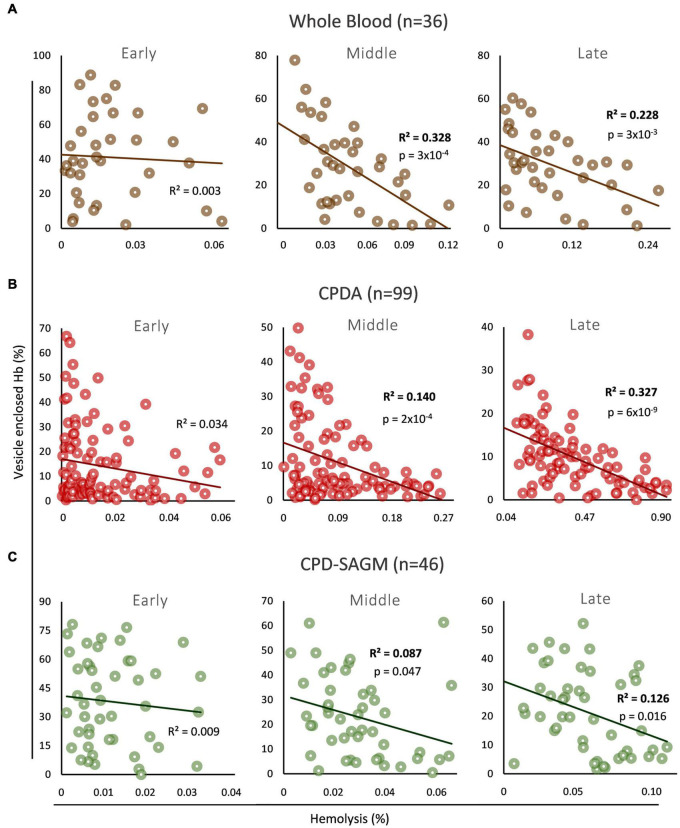
Correlation between vesicle-enclosed hemoglobin (Hb) and storage hemolysis in blood bank conditions. Scatter plots showing the correlation between total free and vesicle-enclosed Hb during early, middle and late storage in **(A)** whole blood, **(B)** CPDA, and **(C)** CPD-SAGM units.

We firstly explored the association (if any) between the levels of total free and vesicle-encapsulated Hb in three distinct storage strategies (whole blood, non-leukoreduced RBCs in CPDA, leukoreduced RBCs in CPD-SAGM) and storage time periods (early, middle, and late storage) (Figure 1). In early storage the two variables did not correlate with each other in any storage strategy, while an inverse correlation between them was evident in both middle and late stored RBCs in whole blood (Figure 1A), non-leukodepleted CPDA (Figure 1B) or leukodepleted CPD/SAGM units (Figure 1C). Overall, it seems that Hb encapsulation inside EVs has a low to moderate (∼9–32%, based on the *R*^2^ values in all conditions mid-storage onward) but nonetheless statistically significant effect upon the total, storage hemolysis levels of middle and late stored RBCs. It should be noted that we observed the lowest effects (9–13%) in CPD/SAGM RBC units.

**FIGURE 2:**
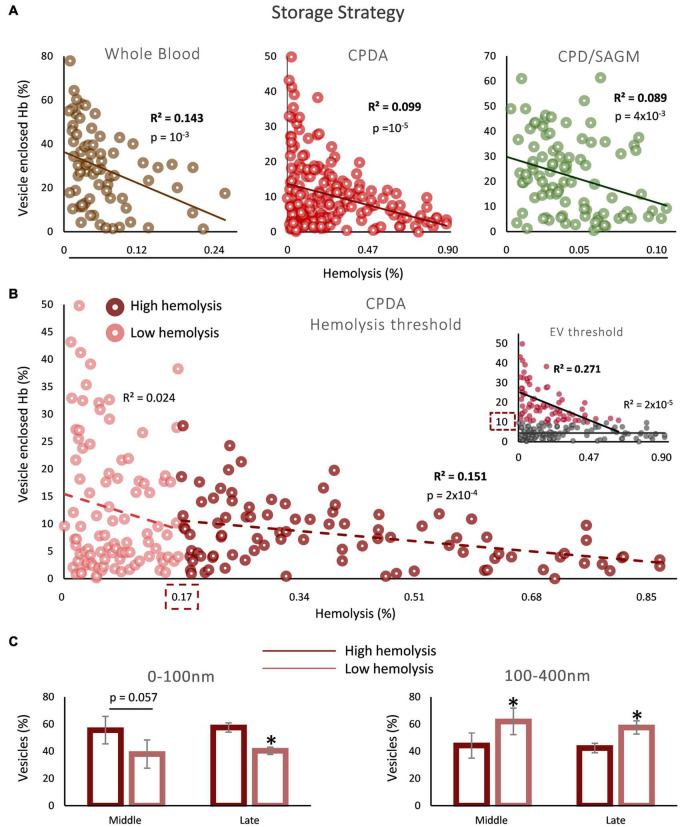
Correlation between vesicle-enclosed Hb and storage hemolysis in blood bank conditions. **(A)** Scatter plots showing the correlation between total free and vesicle-enclosed Hb in whole blood, CPDA and CPD-SAGM units, from mid-storage onward. **(B)** Threshold analysis in CPDA blood units. The dashed boxes indicate the hemolysis or EV-Hb percentage (insert) values that serve as cutoffs. **(C)** Dynamic light scattering (DLS) analysis in selected CPDA samples of high and low hemolysis (above and below the threshold, respectively; *n* = 5 per group) during middle and late storage (horizontal axis). *p < 0.05, high vs low storage hemolysis.

Since the inverse correlation between the percentages of storage hemolysis and EV-Hb was detected in mid-storage onward, we next analyzed the statistical dependability of the two parameters in the sum of samples of middle and late storage groups. Such an analysis serves as the first step, to subsequently check for a potential threshold of hemolysis or percentage of Hb enclosed in EVs that determines the area of the association we found. Indeed, the regression analyses in all storage strategies exceeded the threshold of statistical significance (Figure 2A). After searching for a value above or below which this correlation is observed, it was found that storage hemolysis of 0.17% serves as a potential cut-off, above which this inverse correlation between hemolysis and Hb EV content is evident in non-leukoreduced CPDA units (Figure 2B). No hemolysis threshold was determined for whole blood and CPD/SAGM units. Moreover, since we studied two distinct parameters, we also performed the opposite analysis: we looked for a possible EV-Hb threshold, above or below which the correlation with hemolysis exists. Two percentages stood out: 10 and 30% of vesicle enclosed Hb in CPDA (Figure 2B, insert) and whole blood units (*R*^2^ = 0.202, *p* = 0.009), respectively; above these values the inverse correlation was statistically significant. Again, we found no threshold regarding CPD/SAGM stored RBCs. Finally, we were intrigued to check what would be the outcome of the correlation analysis in case of removing from the model the samples that did not participate in any of the regression lines determined in the two different cut-off analyses, namely CPDA units presenting hemolysis <0.17 and <10% EV-Hb simultaneously. Impressively, an inverse correlation with *R*^2^ = 0.390 and *p* = 10^–15^ was revealed.

To better understand the EV physiology between the two sides of the hemolysis cutoff value, CPDA samples with hemolysis levels below or above 0.17% (*n* = 5 for each group) were analyzed by dynamic light scattering (Figure 2C). Interestingly, while there was a greater percentage of 0–100 nm EVs in the high hemolysis group during both middle and late storage, the opposite pattern was unraveled regarding EVs in the range of 100–400 nm: there was a higher presence of larger EVs in the low hemolysis group in both time points of storage (Figure 2C).

## Discussion

The currently presented data suggest an ordered way of Hb release during the *ex vivo* aging of RBCs at blood bank conditions. Packaging of Hb (along with other sorted membrane and cytosolic loads) into EVs seems to be the mode of choice at lower storage hemolysis levels. These conditions may refer to healthier RBCs, that are able to regulate their membrane vesiculation program and to sacrifice membrane surface area for generating Hb-enclosing EVs, and especially, larger ones. At higher levels of hemolysis insults, however, Hb release seems to follow an erratic process in which the vesicular-free portion of Hb probably prevails, and the Hb-EVs are of smaller size. This finding comes to strengthen the previously reported inverse correlation between hemolysis markers of stored RBCs and the Hb content of released EVs as measured by proteomics analysis in beta-thalassemia minor subjects (Tzounakas et al., 2021b).

This ordered loss of Hb during storage might represent a regulated cellular response to the escalating intensity of the stresses by storage time, especially since the phenomenon is more evident during the middle-late storage period that is characterized by higher levels of stress and accumulation of storage lesions. Indeed, stored RBCs present gradual depletion of energy-related molecules, such as ATP and 2,3-bisphosphoglycerate (D’Alessandro et al., 2020), metabolic rewiring (Reisz et al., 2016), and redox imbalance (Bardyn et al., 2018), that altogether result in cumulative insults to the cytoskeleton, membrane and Hb molecules (Delobel et al., 2016). Having in mind the lack of translation machinery in RBCs, the proteostatic armamentarium, including proteasome, as well as microvesiculation (D’Alessandro et al., 2019) represent the only available working pathways to maintain cell integrity and physiology. Inevitably, the initially discoid stored RBCs irreversibly transform into more rigid shapes, that are prone to lysis, mainly from middle storage onward (Bardyn et al., 2017; Tzounakas et al., 2021b). It was recently proposed that there are two hemolysis pathways related to distinct RBC morphological changes during storage (Melzak et al., 2021). The first one implicates stomatocytes, which transform to spherocytes via endocytic vesiculation (Reinhart and Chien, 1986), while the second implicates echinocytes which turn to spherocytes after shedding vesicles from their spikes (Iglic et al., 2004). It seems plausible to support that RBCs pass through two distinct phases during storage with respect to Hb loss: the first one occurs in the first weeks when RBCs are capable to cope with the energy and redox stresses and respond to them by ostracizing oxidized/denatured Hb molecules mainly via extracellular vesicles (Greenwalt et al., 1991) without losing their integrity; the second one corresponds to middle/late storage when RBCs have already lost a significant part of their volume and membrane/cytoskeletal properties and are more likely to rapture, thus, releasing Hb in a free form rather than sorting it in extracellular vesicles. While senescent RBCs release Hb through membrane vesiculation as part of the *in vivo* aging process (Willekens et al., 2003), during *ex vivo* aging and under storage-related stresses both EVs generation and hemolysis occur. The currently presented hypothesis is also in line with the size distribution of EVs found in the cut-off analysis of CPDA samples as blood units of lower storage hemolysis produce larger EVs (perhaps with the potential to enclose higher proportions of Hb). Thus, these RBCs seem to be competent to sacrifice a part of their membrane while disposing of non-functional Hb. Based on this observation, it would be of great interest for future studies to focus on potential associations between storage related variations in the size/volume of RBCs and of Hb-enclosing EVs.

The overall low to moderate correlation between the two phenomena, suggests that the relative distribution of released Hb in soluble and vesicular forms is affected by additional parameters. Hemolysis is known for its multiparametric nature (Reisz et al., 2017) and irregular character, since its heritability score, studied through genome wide association analysis, is close to zero (Page et al., 2021). Moreover, a recent proteomic study in stored RBC membranes and EVs linked the levels of membrane transporters, as well as the binding of proteostatic molecules and immunoglobins to the membrane, with the Hb cargo of EVs (Tzounakas et al., 2021b). The release of Hb (either in vesicles or as a free molecule) has been also associated with a variety of protein biomarkers, pertinent to the categories of antioxidants and metabolic enzymes (D’Alessandro et al., 2016). Naturally, hemolysis and vesiculation are affected by the storage strategy and component production of choice (Almizraq et al., 2018). The weaker correlation outcome in the leukoreduced CPD/SAGM units compared to the other storage conditions implies the influential effect of mannitol – known for its antioxidant and membrane-protective effects (Hess, 2006) – and of residual WBCs and platelets on the distribution of Hb in vesicles. Of note, whole blood filtered red cell concentrates contain higher numbers of EVs when compared to red cell filtered ones (Almizraq et al., 2018). In addition, it would be of great interest to perform a respective correlation analysis in irradiated blood products that are known to extensively hemolyze, in such a degree that changes the maximum allowed storage time, while at the same time present significant amounts of RBCs with echinocytic morphology and heterogenous size distributions (Lopez-Canizales et al., 2021). In the same context, the narrow range of storage hemolysis variation in the units of whole blood and CPD/SAGM RBCs (attributed probably to the presence of plasma or mannitol), in contrast with the wide range observed in the CPDA units may justify the inability to determine a threshold in the first two groups of units. To support, even though a direct comparison between different storage strategies is not 100% possible in terms of correlation, (a) only a few whole blood and CPD/SAGM RBC units exceeded the currently reported (in CPDA-1 RBC units) hemolysis threshold rendering the determination of possible cut-off points very difficult and, (b) if such a threshold is dictated by the degree of RBC integrity as in our hypothesis, it is logical to acknowledge that the transition from the one way of Hb loss to the other cannot be widely achieved in extremely low hemolysis levels when the structurally competent RBCs preserve their main physiological characteristics. Nevertheless, another factor that might contribute to the inability to find a cut-off value in all three conditions, is the fewer units included in the CPD/SAGM and whole blood groups – a limitation of the present study.

Our findings provide evidence regarding the fate of Hb during RBC accelerated aging and the possible presence of thresholds that might represent the critical point after which ordered survival pathways give their place to irregular rapture. Apart from the quantitative side, which currently represents the gold quality criterion for storage systems, storage hemolysis has qualitative aspects as well, in respect to the differential Hb distribution in vesicles as a function of the storage time. This finding, along with the observed heterogeneity in the composition of RBC-derived micro- and nano-particles (Bosman et al., 2008) and their function as biological response modifiers, suggest a possible differential clinical impact to the recipient. It is known that microparticles containing even low amounts of Hb can reduce nitric oxide bioavailability and potentially affect vasodilation as in the case of free Hb (Liu et al., 2013). However, vesicles represent organized structures with the potential to interact with recipient cells and modify several processes and response phenotypes through both surface molecules and internal loads (Meldolesi, 2018). To what extent and which biological pathway their differential Hb cargo may direct their bioactivity remains to be determined. On this basis, more targeted research on the mechanistic basis of these physiological procedures in additional storage strategies, blood manufacturing methods and distinct donor groups will help to elucidate the extent of the observed associations.

## Data Availability Statement

The raw data supporting the conclusions of this article will be made available by the authors, without undue reservation.

## Ethics Statement

The studies involving human participants were reviewed and approved by Research Bioethics and BioSecure Committee of the Department of Biology, NKUA. Written informed consent for participation was not required for this study in accordance with the national legislation and the institutional requirements.

## Author Contributions

VT and MA designed the study. VT and AA performed the hemolysis experiments, analyzed the results, and prepared the figures. ML analyzed the DLS data. KS and AK were responsible for the sample acquisition and the preparation of RBC units. VT, AA, and MA wrote the first draft of the manuscript. ML, EP, KS, IP, and AK critically commented on the interpretation of data, drafted the manuscript, and contributed to the final version. All authors contributed to the article and approved the submitted version.

## Conflict of Interest

The authors declare that the research was conducted in the absence of any commercial or financial relationships that could be construed as a potential conflict of interest.

## Publisher’s Note

All claims expressed in this article are solely those of the authors and do not necessarily represent those of their affiliated organizations, or those of the publisher, the editors and the reviewers. Any product that may be evaluated in this article, or claim that may be made by its manufacturer, is not guaranteed or endorsed by the publisher.
